# Use of child restraint system and patterns of child transportation in Riyadh, Saudi Arabia

**DOI:** 10.1371/journal.pone.0190471

**Published:** 2018-01-02

**Authors:** Mohammd Alsanea, Emad Masuadi, Tarek Hazwani

**Affiliations:** 1 College of Medicine, King Saud bin Abdulaziz University for Health Sciences, Riyadh, Saudi Arabia; 2 Department of Medical Education, College of Medicine King Saud bin Abdulaziz University for Health Sciences, Riyadh, Saudi Arabia; 3 Pediatric Intensive Care Unit, King Abdullah Specialist Children’s Hospital, Riyadh, Saudi Arabia; University of Illinois at Urbana-Champaign, UNITED STATES

## Abstract

**Objective:**

Child restraint system (CRS) is designed to protect children from injury during motor vehicle crash (MVC). However, there is no regulation or enforcement of CRS use in Saudi Arabia. This study estimated the prevalence of CRS use and identified patterns of child transportation in Riyadh, Saudi Arabia.

**Methods:**

In this cross-sectional study, a self-administered questionnaire was distributed across Riyadh targeting families who drove with children aged less than 5 years. The questionnaire inquired about CRS availability, patterns of child transportation if a CRS was unavailable, seat belt use by the driver and adult passengers, and the perception of CRS.

**Results:**

Of 385 respondents, only 36.6% reported the availability of a CRS (95% CI: 31.8–41.7%), with only half of those reported consistent use 74 (52.2%). Nearly 30% of all children aged less than 5 years were restrained during car journeys. Sitting on the lap of an adult passenger on the front seat was the most common pattern of child transportation (54.5%). Approximately 13.5% of respondents were involved in an MVC while driving with children; 63.5% of these children were unprotected by any safety system. Seat belt use by drivers was low, with only 15.3% reporting constant use.

**Conclusion:**

The prevalence of CRS use in Riyadh is low, and safety practices are seldom used by drivers and passengers. In addition to legal enforcement of CRS use, implementation of a child transportation policy with age-appropriate height and weight specifications is imperative.

## Introduction

Infants and children are not small adults and are unsuited to car seat belts that provide the appropriate protection for adults. They differ from adults in many respects, such as anatomic proportions (the head accounts for a larger proportion of the body), bone maturity, and the locations of vital organs [1].

Child restraint system (CRS) and booster seats are portable seats specifically designed to protect children from injury in a motor vehicle crash (MVC). Research on the efficacy of CRS and booster seats have found that their use is associated with a reduction in the risk of lethal injury of 71–90% in infants (less than 1 year old) and 54% in toddlers (1–4 years old) when passengers involved in an MVC [2,3]. In 2011, the United States (US) National Highway Traffic Safety Administration conducted the National Child Restraint Use Special Study to measure the prevalence of CRS use and drivers’ understanding and attitudes toward the installation of CRS. The results showed that 94% of children (birth to 8 years old) were restrained in CRS or booster seats during car journeys. However, improper installation of CRS was evident in 46% [4]. The use of CRS is mandatory by law in over 90 countries, but only 53 have appropriate specifications in terms of age, weight, and height for CRS use [5].

Although CRS and seat belts have received significant attention in recent decades, road-related injuries (in motorists, pedestrians, cyclists, and other road users) are responsible for the greatest loss of disability adjusted life years in Saudi Arabia [6]. Saudi Arabia has one of the highest rates of road injury and the mortality and morbidity associated with it, with an estimated mortality rate of 27.4 per 100,000 people compared with 10.6 in the US and 2.9 in the United Kingdom [5]. According to the Institute for Health Metrics and Evaluation, MVC alone account for 7.6% of the total deaths reported in all age groups in Saudi Arabia, and the estimated mortality rate of children less than 5 years of age was 4.09 per 100,000 [7].

Although Saudi Arabian law states that the use of CRS is mandatory, there is no enforcement to ensure compliance [5]. Moreover, there is no national data to illustrate the prevalence of CRS use in Saudi Arabia, and there are no national specifications in terms of age, weight, and height to ensure children are using the appropriate seat. This study estimated the prevalence of CRS use in Riyadh, Saudi Arabia, evaluated parents’ perceptions about CRS, and identified patterns of child transportation when a CRS is not used.

## Methods

In this cross-sectional study, a self-administered questionnaire was distributed in multiple locations in Riyadh, including hospitals, shopping malls, and grocery stores, to minimize selection bias. The target group comprised parents or their siblings who drove with children less than 5 years old. We estimated a sample size of 377 respondents using Raosoft^®^ (http://www.raosoft.com/samplesize.html) with a 5% margin of error, 95% confidence interval (CI), and assuming the prevalence of child restraint system CRS to be 50%.

The questionnaire comprised 20 questions on demographic information, safety practices, and perception of CRS. As a secondary objective, we used a five-point Likert scale for car safety belt use (never, rarely, sometimes, often, and always) for driver and passenger regardless of seating location i.e. front seat or back seat. It should be emphasized that all the drivers were men: all women were passengers, because only men are permitted to drive in Saudi Arabia at the current time. We used a five-point Likert scale (strongly disagree, disagree, neutral, agree, and strongly agree) that contained three reverse-worded questions to assess respondents’ perceptions of CRS. The questionnaire was developed by the authors in an Arabic original version. Translation to English and back translation were performed by two different linguists.

The prevalence of CRS use was estimated among families who drove with children less than 5 years old. According to CRS recommendations by the American Academy of Pediatrics (AAP), all children should ride in a CRS until they reach 4 feet 9 inches in height and 8–12 years of age [2]. Because of different practices and perceptions related to driving safety in Saudi Arabia, we used 5 years of age as the cutoff to estimate the prevalence of CRS use. Data collection was started after approval from the Institutional Review Board of King Abdullah International Medical Research Center, Riyadh, Saudi Arabia. Participants were handed a written questionnaire with an attached consent form. The reliability of the perception section was assessed using Cronbach’s alpha in a sample of 55 individuals yielded a score of 0.71. The sample was excluded from the original sample.

### Data analysis and management

Data analysis was performed using the statistical package IBM SPSS Statistics 23.0 (IBM Corp., Armonk, NY, USA). Qualitative variables are presented as frequencies and percentages. Quantitative variables are presented as mean ± standard deviation. Point and interval estimates are presented for the prevalence of CRS availability. The Chi-squared test was used to assess the associations between CRS availability and seat belt use as well as with the demographic variables (gender, age, education level, relationship to the child, number of children aged less than 5 years, monthly income of household). Also, it was used to assess the association between gender and seat belt use. All tests were conducted at a level of significance of 5%.

## Results

Of 440 questionnaires distributed between December 2016 and February 2017 among families with children less than 5 years old, 385 responses were received (a response rate of 87%). The mean age of the respondents was 32.1 ± 7.1 years; 241 (62.6%) were men. The mean family size was 5.2 ± 2.5. The fathers, mothers, and sibling respondents were 198 (51.4%), 94 (24.4%), and 58 (15.5%), respectively.

Regarding CRS availability, 141 (36.6%) of respondents with children less than 5 years old answered that a CRS was available (95% CI: 31.8–41.7%). Of these respondents, only 74 (52.2%) reported they always used it. The total number of children aged less than 5 years in the families surveyed was 554, of which restrained children accounted for only 166 (30%). Of these restrained children, 11 (6.6%) were less than 1 year old, 52 (31.3%) were 1 year old, 48 (29%) were 2 years old, 31 (18.7%) were 3 years old and 24 (14%) were 4 years old.

Demographic variables such as gender, age and number of children aged less than 5 years showed no statistical associations with CRS availability. However, education level and monthly income did show statistical associations with CRS availability (P = 0.004 and 0.045, respectively). (Table 1)

**Table 1:** Baseline characteristics of respondents and its association with child restraint system availability.

				Child restraint system availability				P-value*
				Yes		No		
		*n*	%	*n*	%	*n*	%	
**Gender**	Male	241	62.6	95	39.4	146	60.6	0.141
	Female	144	37.4	46	31.9	98	68.1	
**Age (years)**	< 30	137	36.1	47	34.3	90	65.7	0.091
	30–39	182	47.9	77	42.3	105	57.7	
	40 +	61	16.1	17	27.9	44	72.1	
**Education level**	Up to secondary school	83	21.6	18	21.7	65	78.3	0.004*
	Bachelor	245	63.6	97	39.6	148	60.4	
	Higher education	57	14.8	26	45.6	31	54.4	
**Relationship to the child**	Father	198	51.4	84	42.4	114	57.6	0.013
	Mother	94	24.4	30	31.9	64	68.1	
	Sibling	58	15.1	12	20.7	46	79.3	
	Others	35	9.1	15	42.9	20	57.1	
**Number of children aged less than 5 years**	1	250	64.9	86	34.4	164	65.6	0.215
	2	109	28.3	45	41.3	64	58.7	
	3	18	4.7	5	27.8	13	72.2	
	4	8	2.1	5	62.5	3	37.5	
**Monthly Income of household (Saudi Riyal)**	< 5,000	32	8.4	6	18.8	26	81.2	0.045*
	5,000–9999	121	31.7	39	32.2	82	67.8	
	10,000–14,999	116	30.4	44	37.9	72	62.1	
	15,000–20,000	57	14.9	26	45.6	31	54.4	
	> 20,000	56	14.7	26	46.4	30	53.6	

* Calculated using Chi-squared test

Regarding car safety belt use by adults, 59 (15.3%) reported they always used it and 64 (16.6%) reported they used it often. Never using a safety belt was reported by 84 (21.8%). Women seat belt use was less than men (P-value < 0.001). (Table 2)

**Table 2:** Association between seat belt use and gender.

		Gender				Total		P-value
		Male		Female				
		*n*	%	*n*	%	*n*	%	
**Seat belt use**	Never	23	9.5	61	42.4	84	21.8	<0.001
	Rarely	55	22.8	33	22.9	88	22.9	
	Sometimes	64	26.6	26	18.1	90	23.4	
	Often	51	21.2	13	9.0	64	16.6	
	Always	48	19.9	11	7.6	59	15.3	
						385	100	

There was a significant association between car safety belt use and CRS availability (P-value < 0.001). (Table 3).

**Table 3:** Association between seat belt use and child restraint system availability.

		Child restraint system availability				P-value
		Yes		No		
		*n*	%	*n*	%	
**Seat belt use**	Never	16	19.0	68	81.0	<0.001
	Rarely	24	27.3	64	72.7	
	Sometimes	35	38.9	55	61.1	
	Often	34	53.1	30	46.9	
	Always	32	54.2	27	45.8	

**Fig 1:**
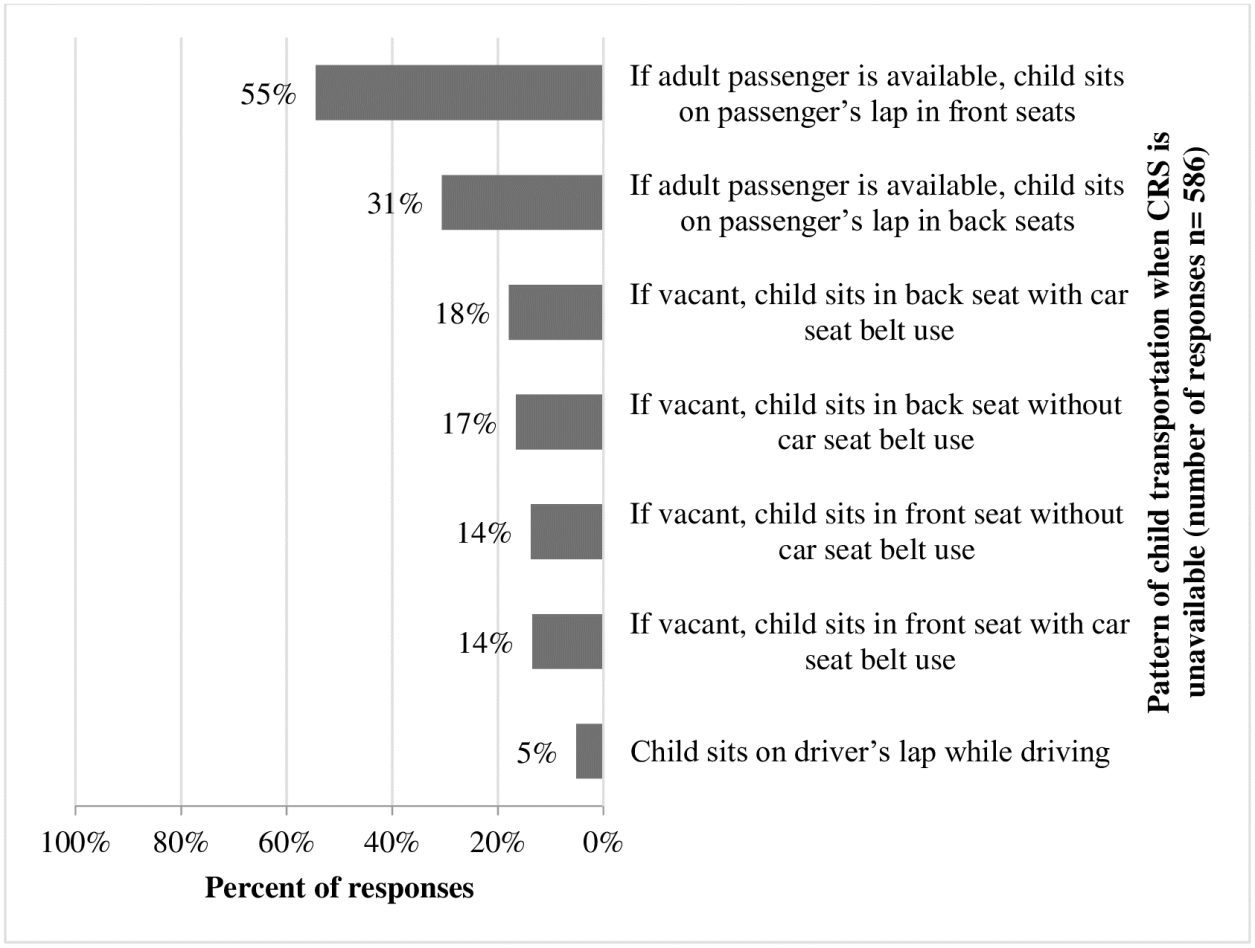
Pattern of child transportation when CRS is unavailable.

A multi-response question about the pattern of child transportation when a CRS was unavailable showed that 210 (54.5%) sat on the lap of an adult passenger in the front seat, and 118 (30.6%) sat similarly on the back seats. Sixty-four (16.6%) and 53 (13.8%) reportedly sat on the back and front seats, respectively, without a car safety belt (Fig 1).

Of all respondents, 52 (13.5%) respondents reported experiencing an MVC while driving with children. No accident-related injuries occurred in 39 (76.5%) children, whereas simple wounds or bruises were reported in 11 (21.6%) children. There was 1 (2%) fracture and one missing data item. No method of child protection was reportedly used in 33 (63.5%) accidents, a car safety belt was used in 10 (19.2%) accidents, and a CRS was used in eight (15.4%) accidents.

**Fig 2:**
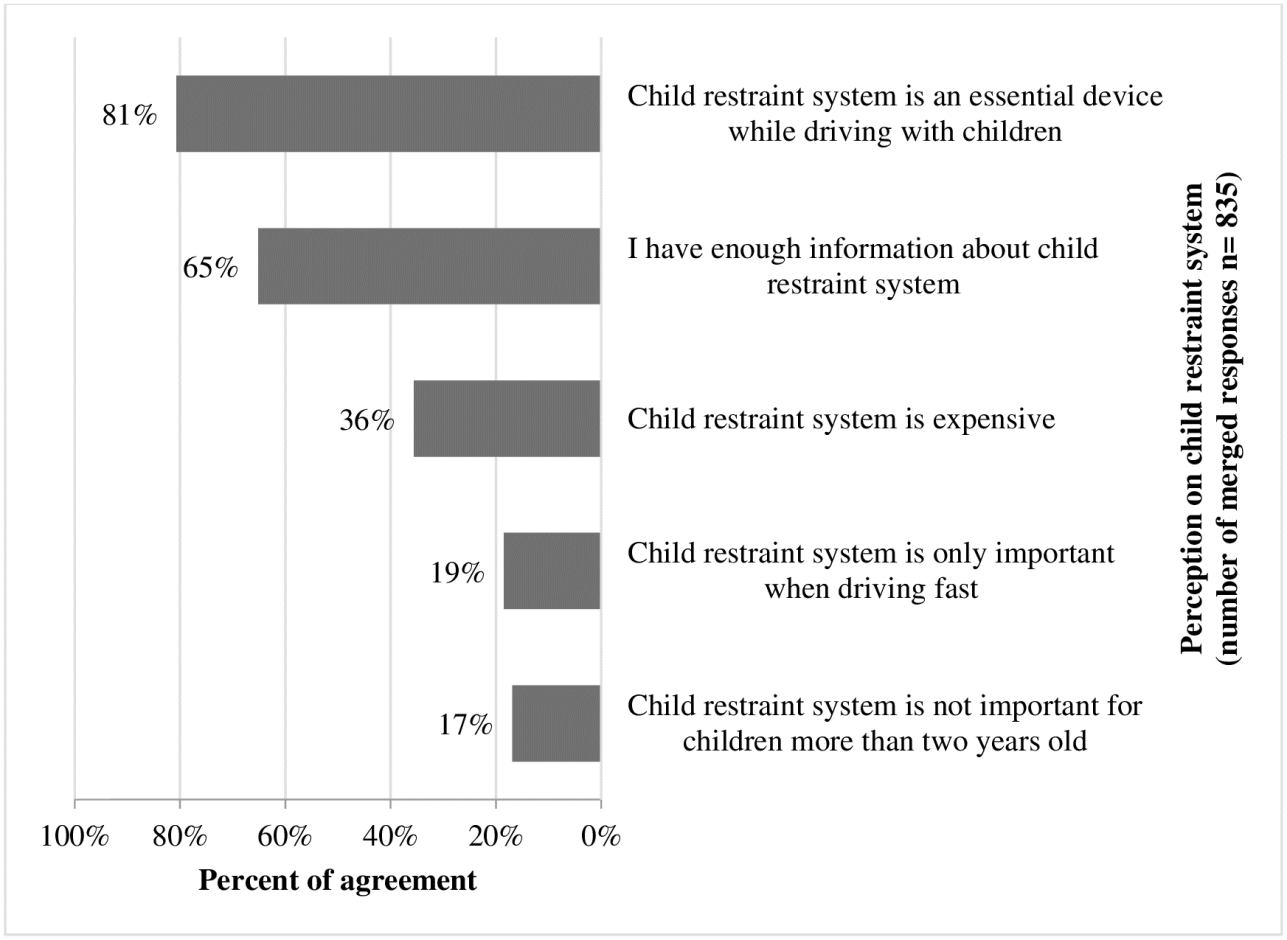
Respondents’ perception on child restraint system CRS. *“strongly agree” and “agree” were merged into one category as level of agreement.

Regarding respondents’ perceptions of CRS, “strongly agree” and “agree” were merged into one category as level of agreement. Three hundred eleven (81%) respondents agreed that CRS is essential while driving with children, and 251 (65%) agreed that they have enough information about CRS (Fig 2).

## Discussion

To the best of our knowledge, this is the first study on CRS use in Riyadh and nationwide. In this study, the prevalence of CRS was low 36.6% with only half of those reported consistent use. Even though we used a lower cutoff point than that recommended by the AAP, just 30% of children aged less than 5 years were restrained by a CRS during car travel. It should be noted that there was no safety or media campaign at the time of data collection that could have manipulated these findings. Although differing in settings and respondents, previous studies have reported CRS use of less than 15% in Turkey and China, 22% in Pakistan, and almost 40% in Brazil [8–11]. In contrast, CRS use exceeds 90% in Australia [12] and the US [4]. Possible reasons for low CRS use include a lack of awareness and inadequate enforcement, particularly where there are no specifications in terms of age, height, and weight to guide the proper use and installment of CRS. In Saudi Arabia, although failure to use a CRS carries a fine of 150 Saudi Riyals (40 US Dollars), there is a lack of official data regarding fines related to CRS unavailability or improper child transportation [13]. All forms of transportation where a CRS is unavailable are inappropriate (Fig 1). Unrestrained children sitting in the front seat is associated with a 40% increased risk of injury if the car is involved in an MVC [14]. In this study, sitting on a passenger’s lap in the front seat was the most commonly reported form of child transportation, and this is associated with a significant risk of injury and hospitalization [15]. Although better education level and income were associated with higher CRS availability, educational or financial limitations should not hinder essential safety practices. Our study reports that higher adult seat belt use is associated with higher CRS use as reported by similar studies [16–18].

A CRS is the most appropriate method to protect children when transporting in a car. However, it is the least used method used when children were involved in an MVC (63.5%). In previous local study, none of the children injured in MVC were restrained [19].

Adult seat belt use was lower than that reported by a local observational study in 2005, which found a 60% use rate [20]. In comparison, adult seat belt use exceeds 80% in several other countries [21–23]. Women reported less seat belt use than men in this study, which contradicts the findings of other studies [22–24]. Although all women are passengers in Saudi Arabia because only men are allowed to drive, we included the option “sometimes” to combine front and back seat use of a seat belt; however “never” was the most common response.

Does self-reported knowledge about CRS positively influence CRS use? This is not what our study found: the knowledge and perception reported by respondents did not match their safety practices. A different model was investigated that used the theory of planned behavior among pregnant women in Saudi Arabia, but failed to show relevance: none of them were observed to have a CRS upon discharge [25]. Although knowledge is an important factor in promoting safety practice, it is not the only issue. Social, environmental, and most importantly behavioral factors are the biggest obstacles when it comes to road safety in Saudi Arabia [26–28].

Evidence-based strategies to promote CRS and seat belt use have already been studied. Strong evidence supports the creation of CRS-related laws with the following characteristics. First, CRS use should be ensured by primary law enforcement, meaning that a driver can be stopped and fined by government officials for improper child safety compliance. This can also be applied to seat belt use, because primary law enforcement of seat belt use is superior to the current secondary law enforcement, through which a driver can be fined for not wearing a seat belt if he is stopped for another road violation [29,30]. Second, CRS use should be governed by age-appropriate laws, where each age group has different specifications. Moreover, there is strong evidence to support the efficacy of the distribution of CRS and education on their use, where parents are offered a CRS through a loan, low-cost rental, or free. This strategy targets parents with financial limitations and a poor understanding of CRS. However, there is insufficient evidence that education-only programs promote CRS use [29]. This is important, because health promotion strategies in our society mainly adopt an education-only approach. Therefore, policymakers and health-care providers must become familiar with the most effective ways of promoting CRS use.

Currently, Riyadh, similarly to other Saudi cities, is a car-centric city when it comes to transportation. With a population of 8 million, children less than 5 years old account for 8.1%, whereas they account for nearly 2.6 million 8.4% of Saudi Arabia [31]. Despite the possibility of self-reporting bias, the findings of this study are suitable for generalization, because Riyadh is the capital of Saudi Arabia: it comprises a heterogeneous society and is the primary target of visitors countrywide for different trades, administrative tasks, and medical purposes.

## Limitations

This study has several limitations. First, the study did not examine the misuse or improper installation of CRS, because this requires inspection rather than reporting. Second, the fact that women do not drive affects the comparability with other studies. Third, self-reporting bias due to the data collection method, although it may not influence over reporting or under reporting of the CRS availability due to the heterogeneity of the sample.

## Supporting information

S1 File Questionnaire in Arabic. (PDF)

S2 File Questionnaire in English. (PDF)
