# Supplementary material for: Use of child restraint system and patterns of child transportation in Riyadh, Saudi Arabia
Source: PLoS One. 2018 Jan 2;13(1):e0190471. doi: 10.1371/journal.pone.0190471 (PMC5749838; doi:10.1371/journal.pone.0190471)
Supplement: S1 File — (PDF) [file pone.0190471.s001.pdf]

نرجو الإجابة على جميع الاسئلة

1. **الجنس** : ذكر ☐ انثى ☐
2. **العمر** : .... سنة
3. **مستوى التعليم:** 1/ أقل من ثانوي☐ 2/ ثانوي☐ 3/ جامعي ☐ 4/ دراسات عليا ☐
4. **صلة القرابة بالأطفال** : 1/ أب☐ . 2/ أم ☐. 3/ أخ او أخت ☐. 4/ أخرى: حدد.........
5. **عدد أفراد الاسرة** : ....
6. **عدد أفراد الأسرة ممن هم أقل من ٥ سنوات:** ....
7. **الدخل الشهري بالريال السعودي**

أقل من ٥،٠٠٠ ☐	٥،٠٠٠ ـ ٩٩٩٩ ☐	١٠،٠٠٠ ـ ١٤،٩٩٩ ☐	١٥،٠٠٠ ـ ٢٠،٠٠٠ ☐	أكثر من ٢٠،٠٠٠ ☐

8. **ما مدى استخدامك لحزام الأمان؟**

1. لا أستخدم ☐ 2. نادرا ☐ 3. بعض الأحيان ☐ 4. معظم الأحيان ☐ 5. دائما ☐

9. **هل يتوفر حاليا الكرسي المخصص للأطفال في سيارتك الشخصية؟**

1. نعم ☐ 2. لا ☐

10. **إذا كانت الإجابة (نعم)، ما مدى استخدامك لكراسي الأطفال؟**

1. لا أستخدم ☐ 2. نادرا ☐ 3. بعض الأحيان ☐ 4. معظم الأحيان ☐ 5. دائما ☐

11. **في حال استخدامك للكراسي المخصصة للأطفال ، ماهو عمر الطفل المستخدم ؟ في حال وجود اكثر من طفل ، اذكر أعمار جميع الأطفال المستخدمين للكراسي المخصصة**

1. اذكر عمر الطفل/الأطفال ....، ....، ....

12. في حال عدم استخدام الكراسي المخصصة للأطفال، ماهي طريقة جلوس الطفل في السيارة؟ بإمكانك اختيار أكثر من اجابه

1. ☐ في حال وجود الأم او من ينوب عنها، يجلس الطفل في حجر(حضن) الراكب في الكرسي الأمامي.
2. ☐ في حال وجود الأم او من ينوب عنها، يجلس الطفل في حجر(حضن) الراكب في الكرسي الخلفي.
3. ☐ في حال عدم وجود احد، يجلس الطفل على كرسي السيارة الأمامي مع استخدام حزام الأمان.
4. ☐ في حال عدم وجود احد، يجلس الطفل على كرسي السيارة الأمامي مع عدم استخدام حزام الأمان.
5. ☐ في حال عدم وجود احد، يجلس الطفل على كرسي السيارة الخلفي مع استخدام حزام الامان.
6. ☐ في حال عدم وجود احد، يجلس الطفل على كرسي السيارة الخلفي مع عدم استخدام حزام الامان.
7. ☐ يجلس الطفل في حجر(حضن) السائق أثناء القيادة.

13. هل سبق وأن تعرضت إلى حادث سير مع وجود طفل مصاحب ؟

1. نعم ☐ 2. لا ☐

- **إذا كانت الإجابة (نعم)، كيف كانت طريقة حماية الطفل ؟**

1/حزام امان السيارة ☐ 2/الكرسي المخصص للاطفال ☐

3/ لايوجد حماية ☐ 4/ اخرى : اذكر ....

- **ماذا حدث للطفل أثناء الحادث ؟**

1/لايوجد اصابات ☐ 2/جروح بسيطة او كدمات ☐ 3/ كسور ☐

4/اصابة حرجة استدعت الدخول للعناية المركزة ☐ 5/ وفاة ☐

الرجاء الإجابة على العبارات التالية:

	غير موافق أبدا	غير موافق	محايد	موافق	موافق جدا
14. لدي معلومات كافية عن كراسي السيارات المخصصة للأطفال	☐	☐	☐	☐	☐
15. تعتبر كراسي السيارات المخصصة للأطفال جزء أساسي في السيارة أثناء التنقل مع الأطفال	☐	☐	☐	☐	☐
16. كراسي الأطفال مهمة فقط إذا كان السائق يقود بسرعات عالية	☐	☐	☐	☐	☐
17. كراسي الأطفال غير مهمة لمن هم فوق سنتين	☐	☐	☐	☐	☐
18. كراسي الأطفال مكلفة	☐	☐	☐	☐	☐
