# Supplementary material for: Use of child restraint system and patterns of child transportation in Riyadh, Saudi Arabia
Source: PLoS One. 2018 Jan 2;13(1):e0190471. doi: 10.1371/journal.pone.0190471 (PMC5749838; doi:10.1371/journal.pone.0190471)
Supplement: S2 File — (PDF) [file pone.0190471.s002.pdf]

Please answer all the following:

1. **Gender:** Male ☐ Female ☐
2. **Age:** ………… years
3. **Education level:** 1/ less than secondary school☐ 2/secondary school☐ 3/ Bachelor ☐ 4/ Higher Study ☐
4. **Relationship to the child:** 1/Father ☐ 2/Mother ☐ 3/Sibling☐ 4/ Other …..
5. **Family member number: …………**
6. **Family member number less than 5 years ……….**
7. **Monthly income in Saudi Riyal**

Less than 5,000 ☐	5,000-10,000 ☐	10,000-15,000 ☐	15,000-20,000 ☐	More than 20,000 ☐

8. **How often do you use car seat belt?**
    1. Never ☐ 2. Rarely ☐ 3. Sometimes ☐ 4. Often ☐ 5. Always☐.
9. **Is child restraint system available in your car at this moment?**
    1. Yes ☐ 2. No ☐
10. **If the answer is (Yes), how often do you use child restraint system?**
    1. Never ☐ 2. Rarely ☐ 3. Sometimes ☐ 4. Often ☐ 5. Always☐.
11. **If you use child restraint system, how old is the child using? if there is more than on child, mention all the ages of children using**
    1. Mention child/children age ………….,…………..,…………

12. If child restraint system is unavailable, how does your child sit in the car? You can pick more than one answer

1. If adult passenger is available, child sits on passenger's lap in front seats☐
2. If adult passenger is available, child sits on passenger's lap in back seats☐
3. If vacant, child sits in back seat with car seat belt use☐
4. If vacant, child sits in back seat without car seat belt use☐
5. If vacant, child sits in front seat without car seat belt use☐
6. If vacant, child sits in front seat with car seat belt use☐
7. Child sits on driver's lap while driving☐

13. Have you ever encountered a car accident while driving with a child?

1. Yes☐. 2. No☐

- **If the answer is (Yes), what was the protection method used for the child?**

  1/ Car seat belt☐. 2/ Child restraint system ☐.

  3/ No protection☐ 4/other☐ ……....

- **What happened to the child during the accident?**

  1/ No injury ☐ 2/ Simple wounds or bruises☐ 3/ Fracture☐.

  4/ Critical injury that required ICU admission☐ 5/ Death☐.

Please answer the following sentences

	Strongly disagree	Disagree	Neutral	Agree	Strongly agree
**14.** Child restraint system is an essential device while driving with children	☐	☐	☐	☐	☐
**15.** I have enough information about child restraint system	☐	☐	☐	☐	☐
**16.** Child restraint system is expensive	☐	☐	☐	☐	☐
**17.** Child restraint system is only important when driving fast	☐	☐	☐	☐	☐
**18.** Child restraint system is not important for children more than two years old	☐	☐	☐	☐	☐
